# Medical Service under the Poor Law

**Published:** 1913-03-01

**Authors:** 


### MEDICAL SERVICE UNDER THE POOR LAW.

### Interview with Dr. Frederic Orton,

FOR FORTY-TWO YEARS POOR-LAW MEDICAL OFFICER FOR HORNSEY.

Considerable attention has lately been devoted to the inefficiency of the Poor-Law service in regard especially to medical treatment. Cases have been cited to show the restricted facilities that exist to enable the very poor to obtain prompt relief in urgent cases. Such restriction is due largely to the cumbrous procedure involved and to the delay that may occur before the case is seen by the parish medical officer.

With a view to obtain some opinions in regard to this matter from a parish doctor who has had a wide experience of Poor-Law cases, our representative called upon Dr. Frederic Orton, who for the past forty-two years has been district medical officer of the Hornsey division of Edmonton. Dr. Orton came to Hornsey at a time when the Poor-Law system of medical relief was much less well organised than it is at present, and when Crouch End was a small hamlet and the population of the district of which "Healthy Hornsey" was the chief town, was below 20,000. To-day the population is nearly 90,000, but Hornsey (to which old inhabitants like Dr. Orton still refer as "the village") has retained its reputation for salubrity, since its death-rate has rarely exceeded nine per thousand, a record of which any town might justly be proud.

Our representative found Dr. Orton quite willing to discuss the various aspects of Poor-Law treatment. He admitted that the ordinary routine involves a certain amount of circumlocutory procedure which in certain cases does involve considerable delay. As suggestions for an improvement he favoured the appointment of additional relieving officers and the extension of the powers of the local authorities.

"My own district is comparatively small, with a radius of about a mile. The Edmonton Infirmary, where our Poor-Law patients who do not get home treatment go to, is about four miles distant and is in charge of a superintendent and assistants. My own work lies principally among local patients who claim parish relief, and as a matter of fact I have nothing to do with the infirmary. If I find the case is an urgent one or one that demands institutional treatment, I transfer it as soon as possible to the infirmary where it receives such treatment."

"You work, of course, in co-operation with the relieving officer. Do you mind telling me how you have found this system of co-operation, so far as results go, works in your parish?"

"It works on the whole very well. During forty years we have had three different relieving officers, and they have all been men of tact and ability. Speaking purely from a medical point of view I think perhaps that they are liable to be 'taken in' by patients with regard to the seriousness of certain cases. But on the whole they discriminate, and they soon learn to become acquainted with human nature as represented by the parish patient. Their work, however, is such that they can hardly deal promptly with all demands, and this of course occasions delay in some cases. I cannot speak too highly of the excellent work done by our relieving officers and their kindly help to myself."

"What is the ordinary method of procedure followed by a person who urgently wishes to obtain sick relief?"

"It is, of course, the same with us as with other parishes; the patient sends to the relieving officer for an order. If the relieving officer considers the case an urgent one he marks the order in red ink. I may say that the number of such urgency orders is relatively small with us; I should not estimate them at more than, say, 5 per cent. Of course the decision as to what is an urgent case rests in the first place with the relieving officer, and he, in the majority of cases, relies on what he is told by the patient or the patient's friends. You know how conditions of urgency are often alleged in the case of private patients; well, the same holds good with regard to parish patients. A man, for instance, will run up and say that his child is dying; when you reach the home you find that the child is suffering from an ordinary uncomplicated attack of measles. The relieving officer has no expert knowledge to enable him to estimate the correct degree of urgency; in each case he has to rely on his experience and on what he is told."

"Do you not think that such a system often entails serious risks to patients in so far as an urgent case may be overlooked or its immediate urgency disregarded?"

"Well, hardly that. I really can't recall an instance where an urgent case has been overlooked in my own parish. Of course, such cases may be overlooked, but I do not think mistakes are frequent. One or two cases I can recall in which an operation of urgency was demanded, and these cases were attended to on the spot." As an instance Dr. Orton cited the case of a patient eighty-three years of age on whom he performed an operation for strangulated femoral hernia, "with a colleague holding the candle to enable me to see." The patient recovered rapidly, and the report of the case, interesting from several aspects, was subsequently published in the *Lancet*.

We pointed out that under the existing system patients often find great difficulties in securing treatment owing to the loss of time involved in delays occasioned by the absence of the relieving officer.

"That is true," admitted Dr. Orton. "In fact I hardly know how the relieving officers manage to do their work so well as they are doing it, seeing that they have such an extended sphere of activity. Very often a patient comes to me complaining that he has been unable to find the relieving officer and that he wants immediate relief. In such cases I do not wait for the order; I go at once and take no chances. The relieving officers have too much work as it is, and ought to have deputies or assistants to whom patients can apply in the absence of the relieving officer."

"Now as to necessitous cases, what is the criterion of necessity? What cases do you afford parish relief?"

"If you mean by parish relief, relief apart from purely medical relief, none. I have nothing to do with that part; it is a matter entirely for the relieving officer. Of course it is very common for me to be asked when I go to see, say, a child whose parents are out of work, 'Doctor, can't you give us nourishment?' I always say that they must apply to the relieving officer, and that I can only give them medicine and treatment. In a great number of cases I find that they send for a bottle of medicine once and then do not trouble me any more; the entire reason for their sending for me in the first instance is because they want nourishment. Forty years ago when I came here I found that my predecessor had been in the habit of usurping the duties of the relieving officer in this respect. His argument was, 'Oh, these folk want food, not medicine,' and his invariable prescription used to be a pint of stout and two lb. of gravy beef. That's not my method. I am chary of giving medical comforts of that description except in cases where they are absolutely wanted."

"Do you find that medical relief is requisitioned more commonly now than it used to be?"

"Most decidedly. Formerly nearly all my parish patients were old and infirm people who clung tenaciously to their homes and refused to go to the infirmary. Now the larger part of my parish practice consists of comparatively young married couples with their families. It is an astonishing thing to me to see the way in which these people expect the parish to help them in every direction. I find it no uncommon thing to be called in at a first confinement of the wife of a young men who is an 'out of worker.' Unemployment and thriftlessness largely account for this change, but I should add that something must be ascribed to the indiscriminate and injudicious efforts of district visitors and well-meaning clergymen. To these good folk, as to Lord Dundonald, who as you may recollect wrote a letter to the *Standard* some time ago in which he pleaded earnestly for the unemployed, all those who go on the parish are deserving people. That is by no means my experience, I regret to say, for there is little doubt, unfortunately, that many of them are undeserving in the highest degree."

"Such experience must tend to make your parish work disagreeable?"

"It does, yes. I have always enjoyed my parish work, for I look upon the parish doctor as discharging an honourable function. For forty-two years I have worked here and never had an official complaint. But of late years I confess the work has become more disagreeable. The amount of clerical work, too, has increased out of all proportion, I think, to the good results accruing from the issue of innumerable certificates."

"How has the Insurance Act affected parish work?"

"At first, before the Act came into operation, I thought that it would tend materially to lessen the amount of work. Since it has been in working, however, we have had more work. I do not say that this increase in the number of patients is due to the Act: it may be due to other factors. For instance, we are now in the period when the amount of parish work is largest owing to weather conditions. But it certainly seems as if the tendency of the Act is to increase the work of the parish doctor, though to what an extent we cannot yet say."

"To come back to urgency cases, you of course have the infirmary to fall back upon in case of operations of urgency?"

"Yes. There is still a prejudice against the infirmary, and many of the older patients obstinately refused to go there. The younger generation is not so prejudiced, and I find that where one member of a family has been treated at an infirmary the other relatives are generally willing to go to the institution, since they see that it has proved its usefulness. Operations are nearly always preferably done at the infirmary, where they command moderately good nursing and up-to-date conditions for aseptic treatment. Our practice is to remove the patient as soon as possible in an ambulance to the infirmary. A delay of a few hours sometimes intervenes before the case can be operated upon, but in the circumstances such delay is hardly to be diminished, especially where the infirmary is at some distance from the patient's home."

